# LncRNA–mRNA expression profile and functional network of vascular dysfunction in septic rats

**DOI:** 10.1186/s40001-022-00961-z

**Published:** 2023-01-07

**Authors:** Ye-Chen Han, Zhu-Jun Shen, Yi-Ning Wang, Ruo-Lan Xiang, Hong-Zhi Xie

**Affiliations:** 1 Department of Cardiology, Peking Union Medical College Hospital, Chinese Academy of Medical Sciences and Peking Union Medical College, No.1 Shuaifuyuan, Dongcheng District, Beijing, 100730 China; 2 Department of Physiology and Pathophysiology, Peking University School of Basic Medical Sciences, Beijing, 100191 China

**Keywords:** Sepsis, Aorta, Long non‑coding RNA, Profile

## Abstract

**Background:**

We used microarrays to analyse the changes in long non-coding RNAs (lncRNAs) and mRNAs in aorta tissue in model rats with lipopolysaccharide-induced sepsis and determined the lncRNA–mRNA and lncRNA–miRNA–mRNA functional networks.

**Methods:**

Wistar rats were intraperitoneally injected with lipopolysaccharide, and the lncRNA and mRNA expression profiles in the aorta were evaluated using microarrays. The functions of the differentially expressed mRNAs were analysed using Gene Ontology and Kyoto Encyclopedia of Genes and Genomes pathway enrichment analyses. We then constructed coding/non-coding co-expression and competing endogenous RNA networks to study the mechanisms related to sepsis in rats.

**Results:**

We identified 503 differentially expressed lncRNAs and 2479 differentially expressed mRNAs in the model rats with lipopolysaccharide-induced sepsis. Mitochondrial fission process 1 (MTFP1) was the most significantly down-regulated mRNA. Bioinformatics analysis showed that the significantly down-regulated mRNAs in the sepsis models were in pathways related to mitochondrial structure, function, and energy metabolism. Coding/non-coding co-expression and competing endogenous RNA analyses were conducted using 12 validated lncRNAs in combination with all mRNAs. The coding/non-coding co-expression analysis showed that the 12 validated lncRNAs were mainly regulatory factors for abnormal energy metabolism, including mitochondrial structure damage and aberrant mitochondrial dynamics. The competing endogenous RNA analysis revealed that the potential functions of these 12 lncRNAs might be related to the inflammatory response.

**Conclusion:**

We determined the differentially expressed lncRNAs and mRNAs in the aorta of septic rats using microarrays. Further studies on these lncRNAs will help elucidate the mechanism of sepsis at the genetic level and may identify potential therapeutic targets.

**Supplementary Information:**

The online version contains supplementary material available at 10.1186/s40001-022-00961-z.

## Introduction

Sepsis is a systemic inflammatory response syndrome that can occur following burns, trauma, and surgery. Sepsis may develop into septic shock [1], which is characterised by microcirculation disorders, histiocytic damage, and multi-organ dysfunction [2]. Septic shock is the most common type of shock and has a mortality rate of 40–80% [3–6]. Cardiovascular dysfunction seriously affects the progression of sepsis, and circulatory dysfunctions, including volume depletion, vasodilation, loss of vascular tone, and myocardial depression, are important features of sepsis [7]. Hypotension can cause a decrease in oxygen delivery and organ perfusion, resulting in impaired organ function, and decreased organ perfusion often leads to multiple organ dysfunction, including acute injury to the kidneys and lungs and myocardial dysfunction.

Long non-coding RNAs (lncRNAs), which are non-coding RNAs greater than 200 nucleotides in length, are poorly conserved endogenous RNAs that do not encode proteins but instead regulate gene expression [8, 9]. Several studies have shown that lncRNAs play various roles in inflammatory responses and several diseases, such as cancer and cardiovascular disease [10, 11]. In mice with LPS-induced sepsis, the lncRNA H19 regulates the expression of aquaporin 1 by sponging miRNA-874 and thus is related to septic myocardial function [12]. The lncRNA NEAT1 promotes inflammatory responses in sepsis-induced liver injury through the Let-7a/TLR4 axis [13]. MALAT1 regulates cardiac inflammation and dysfunction caused by sepsis [14]. However, the roles of all lncRNAs in the vascular injury caused by sepsis remain unclear.

In this study, we identified the differentially expressed (DE) lncRNAs and mRNAs in the aortic tissues of LPS-induced endotoxemic rats using transcriptomic microarray analysis. We then performed functional enrichment analysis and annotation to explore the roles of the DE mRNAs in septic vascular injury.

## Materials and methods

### Model preparation and sample collection

Male Wistar rats (average weight, 200–250 g; average age, 8 weeks), obtained from Charles River Laboratories (Beijing, China), were allowed free access to standard chow and drinking water. The rats were randomly placed into two groups that were treated as follows: rats in the control group were administered an intraperitoneal injection of 0.9% saline (2 mL/kg, *n* = 5), and rats in the LPS group were administered an intraperitoneal injection of LPS (L-2880; Sigma-Aldrich, St. Louis, MI, USA) at 10 mg/kg in 0.9% saline (2 mL of a 5 mg/mL preparation, *n* = 5) [15, 16]. At 24 h after LPS injection, the mean arterial blood pressure (MAP) was calculated by non-invasive measurement of blood pressure. The rats were anaesthetised with an intramuscular injection of ketamine (100 mg/kg body weight) and xylazine (5 mg/kg body weight) and euthanised via CO_2_ inhalation. The aortas were isolated, quickly frozen in liquid nitrogen, and stored at − 80 °C until analysis. All experimental procedures conformed to the Guide for the Care and Use of Laboratory Animals (NIH Publication No. 85-23, revised 1996), and the study protocol was approved by the Laboratory Animal Welfare Ethics Branch and Biomedicine Ethics Committee of Peking University (approval no. LA2020343).

### Microarray analysis

We analysed five pairs of aortic tissues collected from the LPS and control groups using microarray and detected the DE lncRNAs and mRNAs (*n* = 5, each group). The Agilent Gene Expression Hybridisation Kit (Agilent Technology Inc., USA) was used for tissue preparation and microarray hybridisation. An Agilent microarray scanner was used to scan the array, and Agilent feature extraction software was used for the analysis. A fold change > 1.5 and a *P*  < 0.05, were set as the thresholds for differential expression [17]. We used the Kyoto Encyclopedia of Genes and Genomes (KEGG) pathway analysis to identify the important signalling pathways containing the DE mRNAs. Gene Ontology (GO) analysis was used to explore the biological roles of the DE mRNAs in the categories of molecular function, biological process, and cellular component. Gene Set Enrichment Analysis (GSEA) was used to compensate for the shortcomings of individual genes in the analysis.

### Protein–protein interaction analysis

To examine the relationships and functions of the DE mRNAs, a protein–protein interaction (PPI) analysis was performed using the STRING database (https://string-db.org/). The top 100 up-regulated and down-regulated mRNAs were visualised using Cytoscape (v3.6.0) with high confidence ≥ 0.7 and hiding disconnected nodes in the network. The significant modules in the network were scored > 9 using the MCODE (Molecular Complex Detection) Cytoscape plugin.

### Microarray validation using quantitative real-time polymerase chain reaction

Total RNA from rat aortic tissue (50 mg) was extracted using 1 mL of TRIzol reagent (Invitrogen, USA). Approximately, 1000 ng of the extracted RNA was reverse transcribed to cDNA using the SuperScript III Reverse Transcriptase Kit (Invitrogen, USA) according to the manufacturer’s instructions. Quantitative PCR (qPCR) was performed using 2 × PCR master mix (Arraystar, USA) and the ViiA 7 qPCR System (Applied Biosystems; Thermo Fisher Scientific, USA). Glyceraldehyde-3-phosphate dehydrogenase (GAPDH) was used as an internal control, and expression was analysed using the 2^ − ΔΔCt^ method.

### LncRNA–mRNA correlation and co-expression analysis

We evaluated the correlations between the DE mRNAs and lncRNAs by analysing a coding/non-coding (CNC) co-expression network. LncRNA–mRNA pairings with Pearson’s correlation coefficients (PCCs) greater than or equal to 0.90 were identified, and the lncRNA–mRNA co-expression network was visualised using Cytoscape (v3.6.0).

### LncRNA–miRNA–mRNA competitive endogenous RNA regulatory network construction

LncRNAs can sponge miRNAs and prevent them from functioning as negative regulators, thus promoting the expression of the target mRNAs. We used miRanda (http://cbio.mskcc.org/miRNA2003/miranda.html) to predict miRNA-binding sites and considered overlapping miRNA-binding sites on lncRNAs and mRNAs as evidence of lncRNA–miRNA–mRNA interactions. TargetScan (http://www.targetscan.org/vert_72/) was used for these analyses. Finally, we constructed a competitive endogenous RNA (ceRNA) network.

### Determination of serum C-reactive protein (CRP) level

Serum CRP levels were measured using an agglutination test kit (Omega Diagnostics, LTD., Scotland, UK) according to the manufacturer’s instructions.

### *Determining *in vitro* aortic vascular reactivity*

The harvested aortic rings were equilibrated under 1 g of resting tension for 60 min. Then, isometric tension, generated by the vascular smooth muscle, was measured using a force–displacement transducer (K30; Hugosachs Elektronik, March, Germany) and was recorded using a PowerLab data acquisition device and Chart v4.2 software (AD Instruments, Ltd., Oxfordshire, UK). After equilibration, arterial ring responsiveness was assessed by measuring the contraction in response to 80 mM KCl. To assess vasorelaxation, the aorta was pre-contracted with 1 μM phenylephrine before measuring the relaxation response to ACh (10^–9^–10^–5^ M).

### Statistical analysis

GraphPad Prism v5.0 (GraphPad Software, Inc., La Jolla, CA, USA) was used for all statistical analyses. Data are presented as the mean and standard deviation. The statistical significance of differences in the microarray data between the two groups was evaluated using Student’s *t*-test. Differences in expression were considered statistically significant at P < 0.05. Pearson’s correlation test was used to assess the relationship between lncRNAs and mRNAs.

## Results

### LncRNA and mRNA expression in the lipopolysaccharide and control groups

In our previous study, we chose rats with an MAP decline of more than 25%–30% as the sepsis models [18]. Therefore, we defined septic shock as a decrease in MAP to approximately 25–30% of the baseline value, indicating significant myocardial depression, leading to low cardiac output and decreased blood pressure [19]. At approximately 24 h after intraperitoneal injection of LPS, we measured the MAP in the rats. The results showed that the LPS-injected rats are good models for studying septic shock and sepsis [15, 20]. Sepsis induced by LPS injection also caused a significant increase in serum CRP levels compared with the control group (Additional file 1: Fig. S1a). In addition, the KCl-induced aortic contraction and ACh-induced relaxation responses were significantly decreased in the arteries of LPS-injected rats (Additional file 1: Fig. S1b, c), suggesting that sepsis was successfully induced and that the aortic tissue was damaged after LPS injection.

We isolated aortic tissues for microarray analysis of lncRNAs and mRNAs. The analysis identified 503 DE lncRNAs, including 307 up-regulated lncRNAs and 196 down-regulated lncRNAs, and 2479 DE mRNAs, including 1304 up-regulated mRNAs and 1175 down-regulated mRNAs, in the LPS group. The top 50 DE lncRNAs and mRNAs in terms of fold changes in expression are shown in heatmaps (Additional file 1: Fig. S2a, b). All the DE lncRNAs and mRNAs are shown in volcano plots (Additional file 1: Fig. S2c, d), and the distribution of the DE lncRNAs and mRNAs across chromosomes is shown in Circos plots (Additional file 1: Fig. S3a, b). Table S1 (See Additional file 1) lists the top 20 DE lncRNAs, and Table S2 (see Additional file 1) lists the top 20 DE mRNAs. The GEO accession number of the dataset used in this study is GSE144439.

### Gene ontology and Kyoto encyclopedia of genes and genomes analyses of the differentially expressed mRNAs

**Fig. 1:**
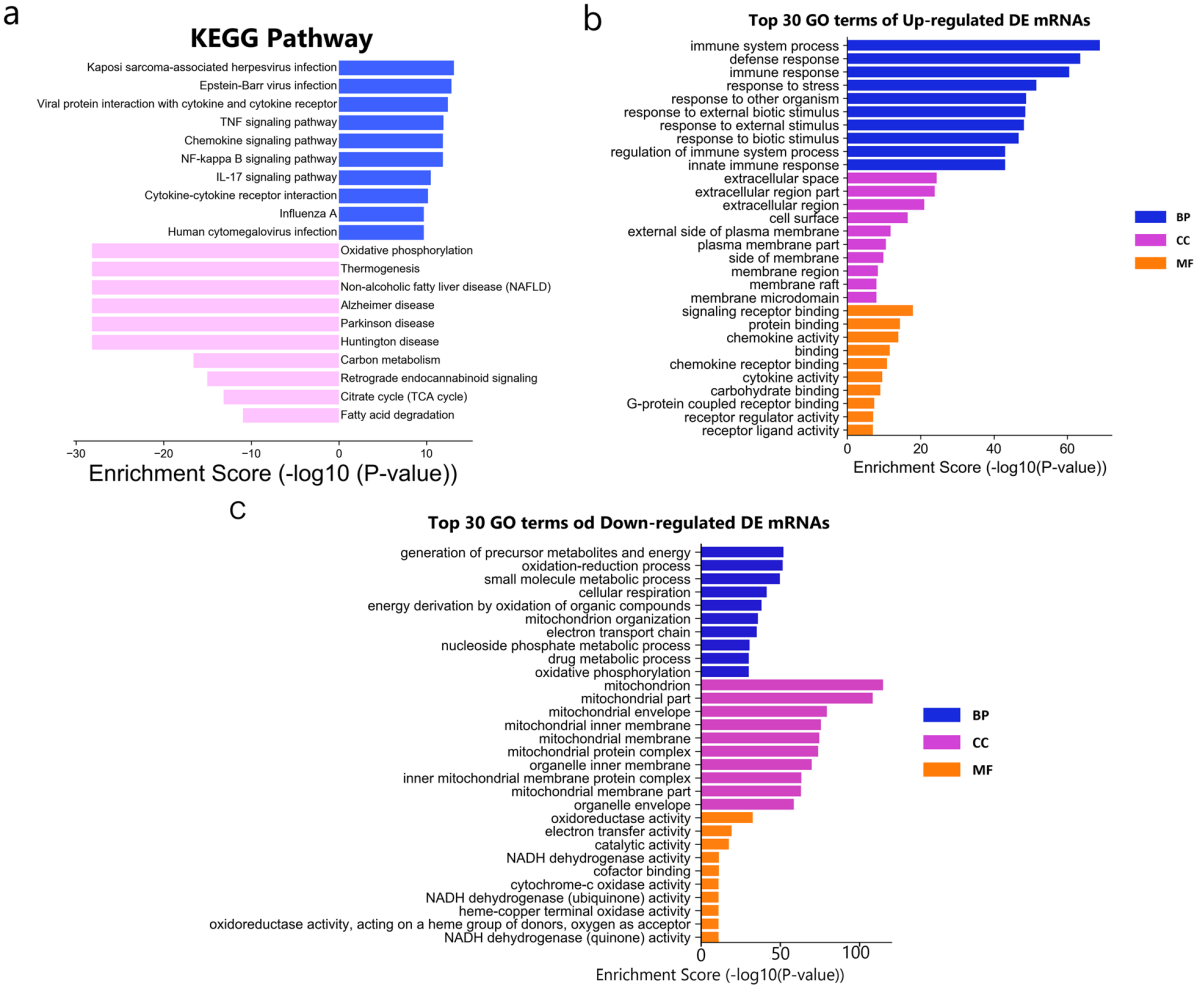
Functional analysis of the differentially expressed mRNAs and lncRNAs. **a** Kyoto Encyclopedia of Genes and Genomes pathway analyses of differentially expressed mRNAs. Blue and pink bar plots represent the Top 10 enriched pathways of up- and down-regulated DE mRNAs, respectively; Pathways related to the research objectives **b** up-regulated and **c** down-regulated DE mRNAs. *DE* differentially expressed

KEGG pathway analysis was performed to explore the pathways involved in sepsis. The analysis revealed that the up-regulated mRNAs in the LPS group were involved in 95 pathways, and the down-regulated mRNAs were involved in 44 pathways. Figure 1a shows that oxidative phosphorylation and thermogenesis were two of the major enriched pathways associated with down-regulated mRNAs, whereas Kaposi sarcoma-associated herpesvirus infection and Epstein-Barr virus infection were two of the major enriched pathways associated with the up-regulated mRNAs. The up-regulated mRNAs were enriched in TNF signalling pathway, chemokine signalling pathway, NF-κB signalling pathway, and IL-17 signalling pathway (Additional File 1: Fig. S4a). The down-regulated mRNAs were enriched in oxidative phosphorylation and the citrate cycle (TCA cycle) (Additional File 1: Fig. S4b).

GO analyses were performed to determine the functions of the DE mRNAs in sepsis. Figure 1b and c show the top 10 GO terms in biological processes, cellular components, and molecular functions. In the LPS group, the GO terms related to the most significantly down-regulated mRNAs were generation of precursor metabolites and energy (biological process), mitochondrion (cellular component), and oxidoreductase activity (molecular function); the terms related to the most significantly up-regulated mRNAs were immune system process (biological process), extracellular space (cellular component), and signalling receptor binding (molecular function).

**Fig. 2:**
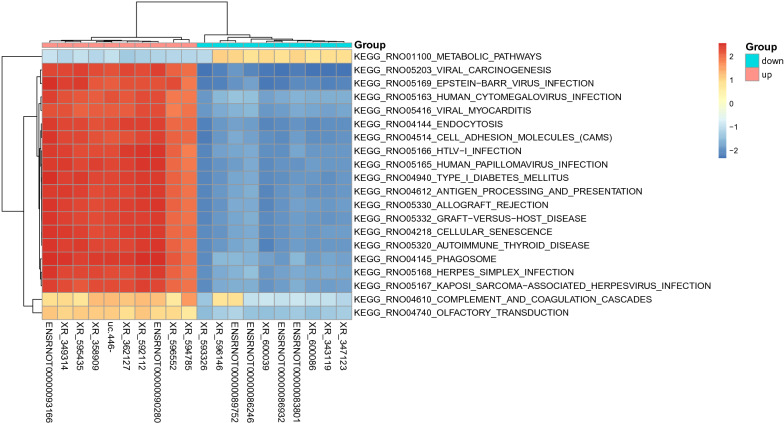
Gene Set Enrichment analysis of Top20 DE lncRNAs. DE, differentially expressed

To determine the relationships among the DE mRNAs, we performed GSEA to identify lncRNA-related pathways. As shown in Fig. 2, the top 20 DE lncRNAs in the same pathway were clustered together. The DE lncRNAs in the LPS group were mainly related to viral carcinogenesis, viral myocarditis, and cell adhesion molecules.

### Protein–protein interaction analysis of the differentially expressed mRNAs

Determining protein–protein interactions (PPIs) are critical for understanding the biological functions of proteins and drug discovery. To determine the relationships among the identified DE mRNAs, we constructed a PPI network. The PPI network containing the top 100 DE mRNAs, which was constructed using Cytoscape (v3.6.0) with high confidence ≥ 0.7 and hiding disconnected nodes, is shown in Fig. S5a (see Additional File 1). The network consisted of 84 nodes with an average of 5.19 neighbours and 218 edges. Using MCODE, a significant module that was filtered out with a score > 9 is shown in Fig. S5b (see Additional File 1).

### Validation of the lncRNA and mRNA microarray results

We performed qRT-PCR to verify the 20 most significant DE lncRNAs (Additional File 1: Fig. S6a). Of the 20 analysed lncRNAs, the results for 14 were consistent with the microarray analysis, five were down-regulated (ENSRNOT00000079309, ENSRNOT00000079370, ENSRNOT00000086932, XR_086340, XR_344417), and eight were up-regulated (ENSRNOT00000077237, ENSRNOT00000089798, XR_349314, XR_349493, XR_362127, XR_592112, XR_592660, and XR_596552). We also performed qRT-PCR to verify the 20 most significant DE mRNAs (Additional file 1: Fig. S6b). The primers used to evaluate the expression levels of the top 20 most significantly DE lncRNAs and mRNAs are shown in Tables S3 and S4 (see Additional File 1), respectively. Among the 20 that were analysed, 12 were consistent with the microarray analysis, four were down-regulated [Cox8b, Lrrc52, mitochondrial fission process 1 (MTFP1), and Nr0b2], and eight were up-regulated (C3, Ccl7, Cxcl1, Il6, Lcn2, Orm1, S100a9, and Sectm1b). Compared to the microarray results, the validation rate of the DE lncRNAs and DE mRNAs was 60–70%. These validated DE lncRNAs and mRNAs were then further analysed.

### Coding/non-coding gene co-expression network analysis and gene ontology and Kyoto encyclopedia of genes and genomes pathway analyses based on the results of the coding/non-coding gene co-expression analysis

**Fig. 3:**
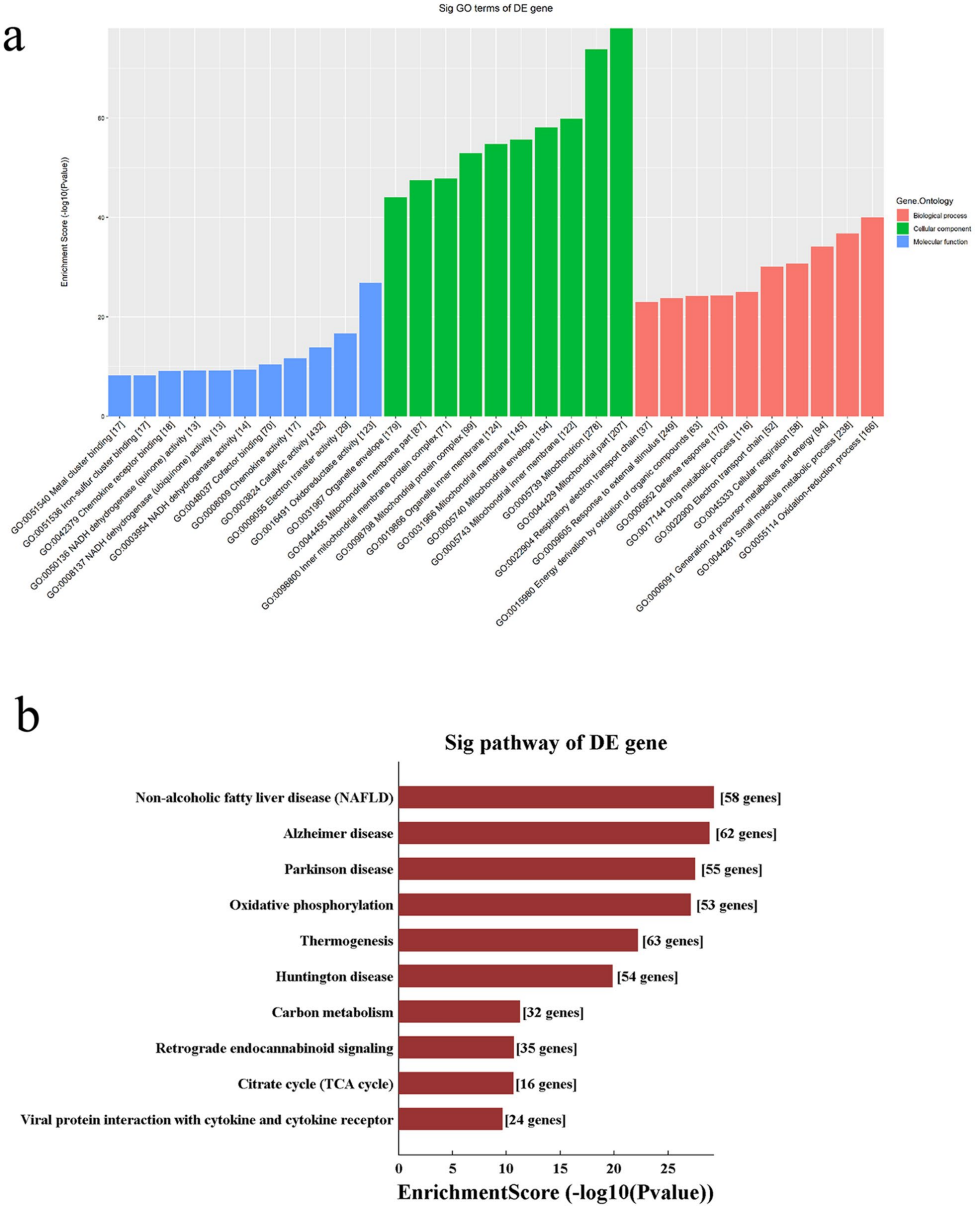
Gene enrichment and pathway analysis of the lncRNAs–mRNAs coexpressed network. **a** Gene Ontology and **b** Kyoto Encyclopedia of Genes and Genomes pathway analyses based on CNC analysis results. CNC, coding/noncoding co-expression

We used the data for the 14 qRT-PCR–verified DE lncRNAs and the normalised data for the DE mRNAs to calculate the correlation coefficients, and selected records of ABS (PCC) ≥ 0.9, P ≤ 0.05, and a false discovery rate (FDR) ≤ 1 for the CNC analysis. We then performed KEGG and GO analyses of the co-expressed target genes. Based on the results of the KEGG and GO analyses, the pathways and enriched genes related to the research targets were screened. The results of the GO analysis are shown in Fig. 3a. The top two enriched biological processes were oxidation–reduction process and small molecule metabolic process; the top two enriched cellular components were the mitochondrial part and mitochondrion; and the top two enriched molecular functions were oxidoreductase activity and electron transfer activity. The KEGG pathway analysis revealed three main pathways: non-alcoholic fatty liver disease (NAFLD), Leishmaniasis, and Alzheimer’s disease (Fig. 3b).

**Fig. 4:**
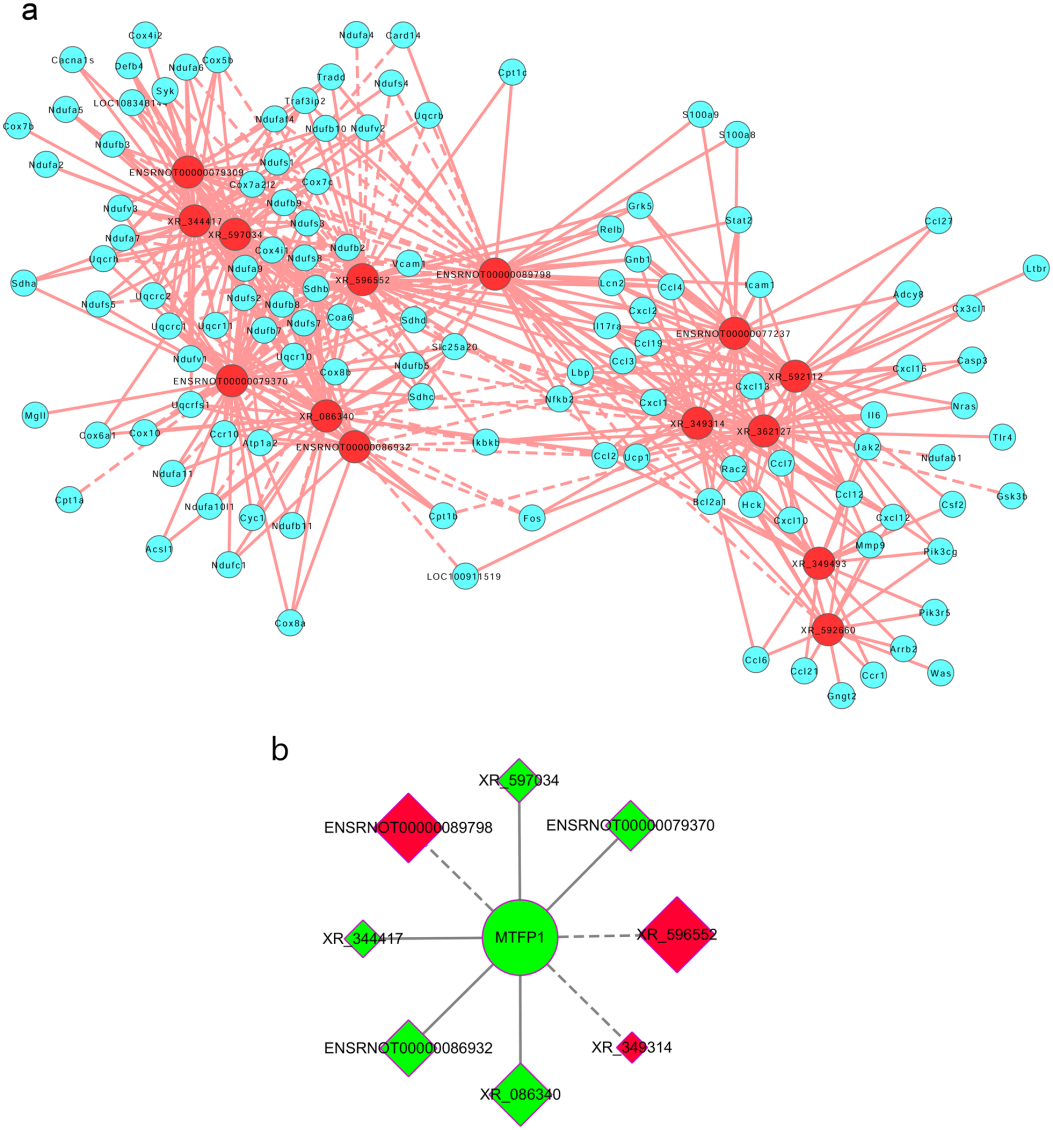
The lncRNA–mRNA co-expressed network of rat aorta tissue in sepsis group compared with control group. **a** DE lncRNAs and DE mRNAs CNC network. Red nodes are lncRNAs, blue nodes are mRNAs; **b** MTFP1 and DE lncRNAs CNC network. Ellipse is MTFP1. Diamonds are lncRNAs. Red and green colors represent up‐ and down-regulation, respectively. Positive correlation is a solid line, negative correlation is a dashed line. *CNC* coding/noncoding co-expression; *MTFP1* mitochondrial fission process 1; *DE* differentially expressed

To construct the co-expression network (Fig. 4a), we chose the genes related to the research topic in the top five enriched KEGG pathways of thermogenesis, chemokine signalling pathway, IL-17 signalling pathway, cardiac muscle contraction, and NF-κB signalling pathway. Among the identified genes, we were most interested in MTFP1. Therefore, we constructed a network containing MTFP1 and its co-expressed DE lncRNAs (Fig. 4b).

### Construction of ceRNA networks and gene ontology and Kyoto encyclopedia of genes and genomes pathway analysis based on the ceRNA network results

**Fig. 5:**
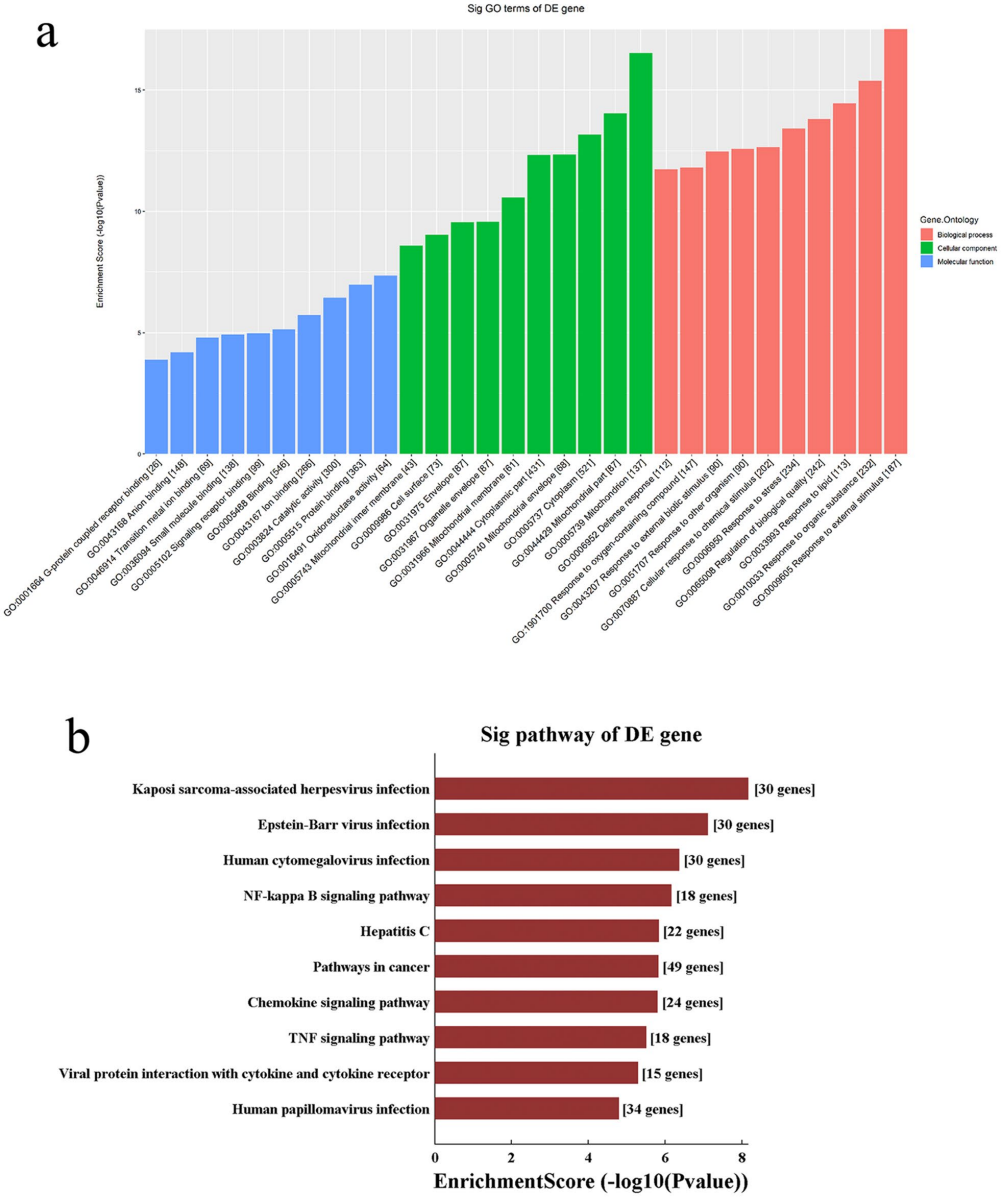
Gene enrichment and pathway analysis of the competing endogenous RNA analysis. **a** Gene Ontology and **b** Kyoto Encyclopedia of Genes and Genomes pathway analyses based on ceRNA analysis results. ceRNA, competing endogenous RNA

**Fig. 6:**
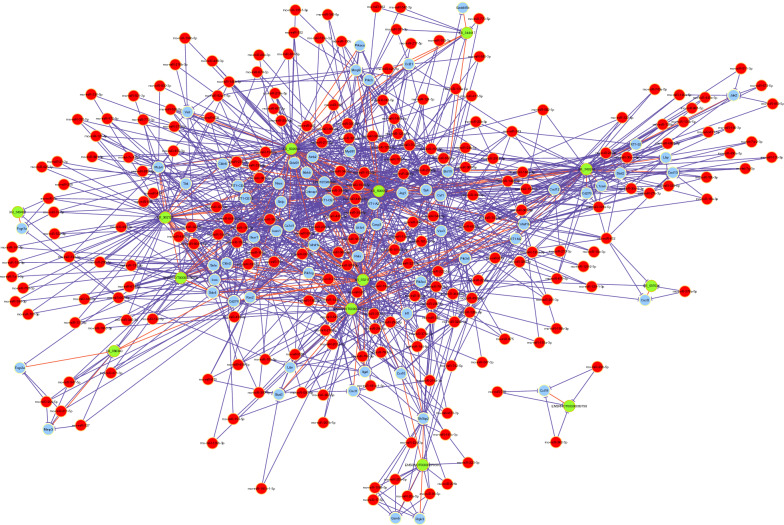
The competing endogenous RNA network of rat aorta tissue in sepsis group compared with control group. DE lncRNAs and DE mRNAs ceRNA network. Red circles represent miRNAs; blue circles represent mRNAs; and green circles represent lncRNAs. *DE* differentially expressed

**Fig. 7:**
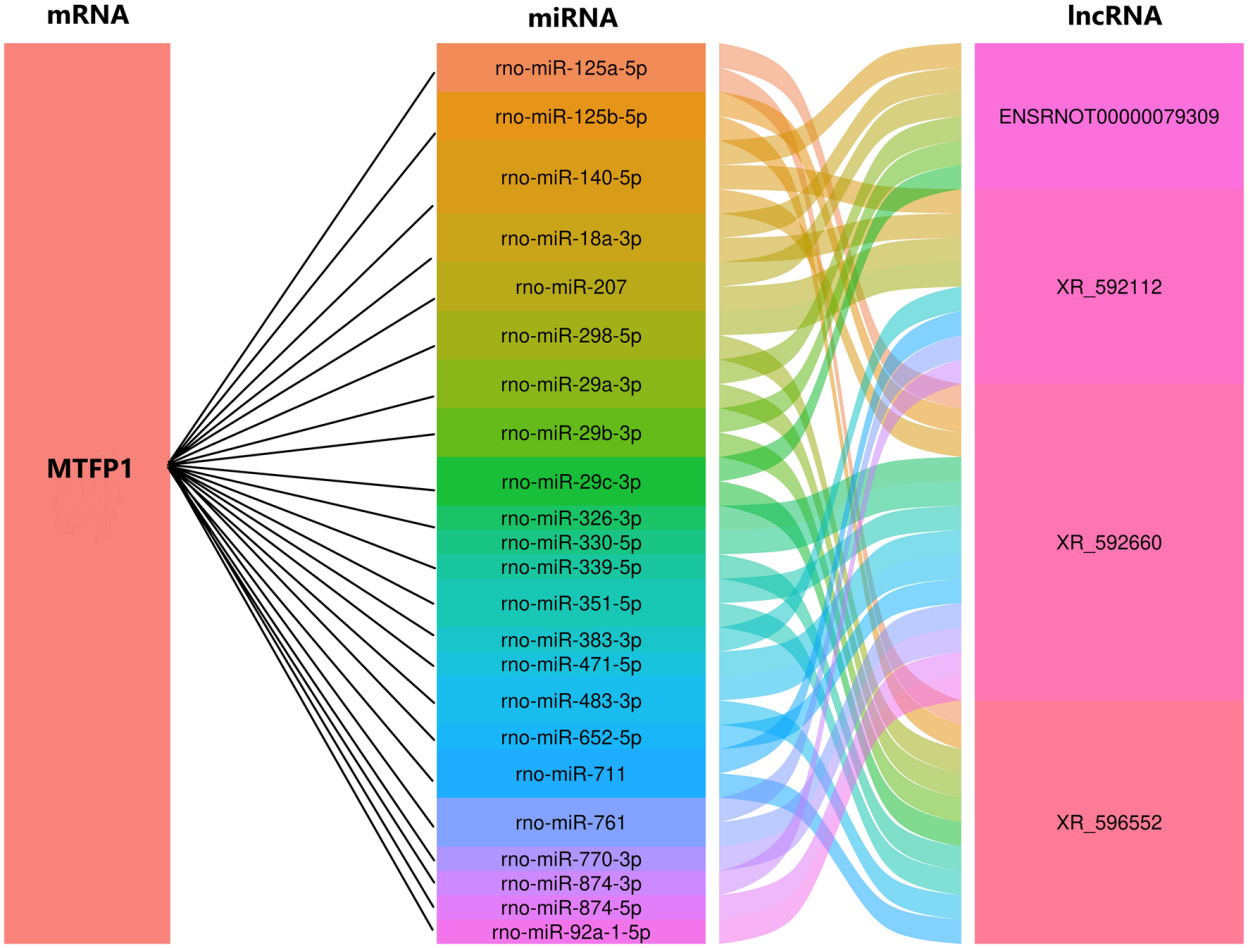
Mitochondrial fission process 1 and DE mRNAs competing endogenous RNA alluvial plot

LncRNAs, which can function as ceRNAs or miRNA sponges, alleviate the inhibitory effects of miRNAs on target genes, thus increasing target gene expression. We used the data from the 14 qRT-PCR-verified DE lncRNAs and DE mRNAs to perform a ceRNA analysis. The number of predicted miRNA-IDs was limited to 1000, and the predicted mRNAs were used to perform KEGG and GO analyses. The results of the GO analysis are shown in Fig. 5a, and the top two enriched biological processes were response to external stimulus and response to organic substance; the top two enriched cellular components were mitochondrion and mitochondrial part; and the top two enriched molecular functions were oxidoreductase activity and protein binding. The KEGG pathway analysis revealed 109 enriched pathways, and the three most important pathways were Kaposi sarcoma-associated herpesvirus infection, Epstein–Barr virus infection, and human cytomegalovirus infection (Fig. 5b). To construct the ceRNA network (Fig. 6), we chose the genes related to the research objectives in five enriched KEGG pathways: NF-κB signalling pathway, chemokine signalling pathway, TNF signalling pathway, natural killer cell-mediated cytotoxicity, and cell adhesion molecules. MTFP1, miRNAs, and the targeted genes are shown in a ceRNA alluvial plot (Fig. 7).

## Discussion

Studies have revealed abnormal expression levels of certain lncRNAs in sepsis-induced organ dysfunction. Therefore, we performed a microarray analysis of the lncRNAs and mRNAs in the aortic tissues of septic model rats to identify the changes in lncRNA and mRNA expression in septic vascular tissue with an aim to provide new targets for improving circulatory failure in sepsis. We identified 503 DE lncRNAs (307 up-regulated lncRNAs and 196 down-regulated lncRNAs) and 2479 DE mRNAs (1304 up-regulated mRNAs and 1175 down-regulated mRNAs).

We first analysed the functions of the DE mRNAs. We found that MTFP1 was the top down-regulated DE mRNA. MTFP1 is a nuclear-encoded protein that promotes mitochondrial fission and is closely related to mitochondrial fission and fusion [21]. Mitochondrial fragmentation often occurs in dysfunctional mitochondria, resulting in decreased ATP production [22]. Increasing evidence indicates that mitochondrial dynamics are associated with mitochondrial metabolism and vascular relaxation induced by mitochondrial inhibition [23, 24]. We observed significant down-regulation of MTFP1 in the aortic tissues of rats with LPS-induced sepsis. This indicates that abnormalities in mitochondrial dynamics occur in sepsis. If these abnormalities cause vascular relaxation, then mitochondrial dynamics could be closely related to the decrease in blood pressure observed in sepsis.

We performed GO and KEGG analyses of the DE mRNAs to determine which signalling pathways and functions may be altered in the aortic tissue in septic shock. KEGG analyses were conducted to identify relevant functional pathways, which showed that among the down-regulated pathways, oxidative phosphorylation was the most enriched pathway, and the citrate cycle (TCA cycle) was also highly enriched. GO analysis was performed to study the functions of the DE mRNAs, which showed that the significantly down-regulated biological processes were generation of precursor metabolites and energy, oxidation–reduction process, cellular respiration, energy derivation by oxidation of organic compounds, mitochondrion organisation, electron transport chain, and oxidative phosphorylation. Nine of the ten most significantly down-regulated cellular components were related to mitochondria (mitochondrion, mitochondrial part, mitochondrial envelope, mitochondrial inner membrane, mitochondrial membrane, mitochondrial protein complex, inner mitochondrial membrane protein complex, and mitochondrial membrane part). The significantly down-regulated molecular functions were oxidoreductase activity, electron transfer activity, NADH dehydrogenase activity, cytochrome-c oxidase activity, NADH dehydrogenase (ubiquinone) activity, oxidoreductase activity, and NADH dehydrogenase (quinone) activity. These results suggest that the most significant changes in the vascular tissue under septic shock are associated with energy metabolism, since many molecules related to energy metabolism and their signalling pathways were significantly down-regulated. Recent studies have indicated that the key pathophysiological mechanism of sepsis is metabolic decline rather than structural damage [7], and ATP levels and the phosphocreatine/ATP ratio were lower in patients with severe septic shock [25]. Abnormal energy metabolism occurs in various tissues in patients with sepsis, and mitochondrial dysfunction has been observed in skeletal muscle, platelets, and peripheral blood mononuclear cells [26, 27]. Mitochondrial dysfunction was also observed in liver cells isolated from healthy humans exposed to endotoxins [7]. GO analysis indicated that mitochondrial structure proteins were significantly down-regulated, suggesting that mitochondrial structural damage plays an important role in metabolic decline. We suspected that as a result of the serious damage to the mitochondrial structure, the biological processes and molecular functions related to the respiratory chain were also significantly altered; oxidative phosphorylation was also significantly altered, ultimately leading to insufficient energy production and vascular function damage. This was the most prominent change observed in sepsis.

Increasing evidence has shown that lncRNAs play vital roles in sepsis-induced organ dysfunction, including vascular tissue damage. Therefore, we conducted a microarray analysis of the lncRNAs in the aortas of model rats with LPS-induced sepsis to identify the relevant lncRNAs. We performed a qRT-PCR validation of the 20 lncRNAs with the most significantly altered expression and selected 12 of the validated lncRNAs for further analysis. We conducted CNC and ceRNA analyses of these 12 lncRNAs. We then performed GO and KEGG analyses of the predicted target genes to identify the molecules and pathways regulated by these DE lncRNAs.

Next, these 12 DE lncRNAs and mRNAs were subjected to CNC network analysis; we first determined the altered mRNAs related to these 12 lncRNAs. The CNC network analysis revealed potential regulatory relationships between mRNAs and these 12 lncRNAs. Interestingly, the predicted mRNAs were highly consistent with the DE mRNA analysis. The KEGG analysis revealed significant enrichment in oxidative phosphorylation and the citrate cycle (TCA cycle). The biological processes oxidation–reduction process, generation of precursor metabolites and energy, cellular respiration, electron transport chain, and respiratory electron transport chain had high enrichment scores. The top nine enriched cellular components were all related to mitochondrial structure. The top three enriched molecular functions were oxidoreductase, electron transfer, and catalytic activities. These results suggest that the most significant DE lncRNAs were involved in energy metabolism. In other words, the regulation of lncRNA levels might be an important mechanism by which sepsis leads to energy metabolism dysfunction in vascular tissue. This indicates that lncRNAs may be key players in sepsis-related vascular damage.

LncRNAs regulate mRNA expression in several ways. One of the primary mechanisms by which lncRNAs alter the expression of downstream mRNAs is by sponging regulatory miRNAs, thus reducing their levels. Therefore, we performed a ceRNA analysis of the 12 lncRNAs and DE mRNAs in the hope of identifying the mRNAs that are regulated by miRNAs. KEGG and GO analyses of these mRNAs revealed that five of the top ten enriched cellular components were related to mitochondrial structure, and oxidoreductase activity was the most enriched molecular function, suggesting that the altered mitochondrial structure was regulated by lncRNA–miRNA interaction. In addition, the KEGG analysis showed that several inflammation-related pathways were highly enriched, including the NF-κB, chemokine, and TNF signalling pathways. These results suggest that although lncRNAs regulate many mRNAs related to energy metabolism, lncRNA–miRNA–mRNA interaction is not the main way. LncRNAs can regulate the mRNAs involved in energy metabolism in other ways. However, lncRNA–miRNA–mRNA interactions may be involved in the inflammatory response. The mechanisms by which lncRNAs regulate mRNAs related to energy metabolism require further study.

The cardiovascular system is essential for maintaining adequate organ perfusion. Therefore, cardiovascular dysfunction has a direct impact on sepsis. Severe circulatory system dysfunction is closely associated with cell metabolism disorders. Multiple organ failure caused by septicaemia is often accompanied by only limited cell death and frequent recovery of organ function, suggesting that metabolic shutdown, rather than structural damage, is the main cause [7]. Blood vessels require well-structured, functional mitochondria to ensure sufficient energy supply, normal physiological function, and adequate organ perfusion. However, in an inflammatory microenvironment, mitochondrial oxidative phosphorylation is disrupted, which reduces the supply of ATP [28–30]. Previous studies have shown aberrant energy metabolism in several organs during sepsis. For example, LPS-induced acute myocardial injury is mainly caused by mitochondrial damage [25]. The decrease in mitochondrial respiration leads to excess ROS generation, causing oxidative damage to cells, which can result in cell death. When ATP is insufficient, a variety of cell death processes can occur, including necrosis, apoptosis, reticulum, pyroptosis, and autophagy-induced cell death, causing organ damage [31]. This reduction in ATP generation leads to a bioenergy deficit. It has long been viewed as a critical factor in sepsis-induced organ failure. Our high-throughput mRNA sequencing revealed significant energy metabolism damage in the aortic tissue, including mitochondria structural damage and aberrant mitochondrial dynamics. We suspect that insufficient energy production in vascular tissue damages smooth muscle cells, directly leading to vascular relaxation and hypotension. Insufficient energy production can also damage vascular endothelial cells, cause disseminated intravascular coagulation, and eventually lead to multiple organ failure [32]. Some studies have shown that energy-related treatments such as β-receptor blockers can improve the outcomes of patients with heart failure [3]. Therefore, drugs that improve energy metabolism may also be helpful in the treatment of septic shock. Our high-throughput lncRNA sequencing showed that lncRNAs are the main regulators of this process and that miRNAs are also involved. Thus, further research is warranted.

This study has some limitations. We tested vascular tissue, but we did not isolate a smooth muscle, endothelial tissues, or other vascular cells. Therefore, we could not state which cell types were altered; thus, more detailed research is needed in the future.

## Conclusions

MTFP1 was the most significantly down-regulated mRNA in the aortic tissue of rats with LPS-induced sepsis. We found that pathways related to mitochondrial structure, function, and energy metabolism were significantly down-regulated in the septic model rats. CNC and ceRNA analysis showed that 12 validated lncRNAs were the main factors regulating abnormal energy metabolism, including mitochondrial structure damage and aberrant mitochondrial dynamics. LncRNA–miRNA–mRNA interactions may be involved in the inflammatory response in sepsis. In addition to expanding our understanding of the pathogenesis of sepsis, our functional lncRNA prediction may provide new treatment targets, such as energy-related targets, which may improve the prognosis of patients with septic shock.

## Supplementary Information

**Additional file 1: Table S1.** The detailed information of the top ten up-regulated and top ten down-regulated lncRNAs. **Table S2. **The detailed information of the top ten up-regulated and top ten down-regulated mRNAs. **Table S3. **Primers for LncRNAs validated by qRT-PCR. **Table S4.** Primers for mRNAs validated by qRT-PCR. **Fig. S1.** Verify the sepsis model. (a) Effect of LPS on serum C-reactive protein. Effect of LPS on in vitro vascular (b) contraction reactivity and (c) relaxation responses. n = 4. * *P* < 0.05, significant from the control and LPS group. LPS, Lipopolysaccharide. **Fig. S2.** Systematic analysis of significantly differentially expressed lncRNAs and mRNA in aorta tissue. Heat maps showing the expression profiles of (a) lncRNAs and (b) mRNAs; Volcano plots presenting differences in the expression of (c) lncRNAs and (d) mRNAs between the LPS and control groups. Values plotted on the x- and y-axes represent the averaged normalised signal values of each group (log2-scaled). LPS, Lipopolysaccharide. **Fig. S3.** The distribution of DE lncRNAs and DE mRNAs on chromosomes. (a, b) Circos plots representing the distribution of DE lncRNAs and DE mRNAs on chromosomes. The outer layer cycle is the chromosome map of the human genome. The inner layers represent the distribution of DE mRNAs and DE lncRNAs on different chromosomes, respectively. Red and green colors represent up- and down-regulation, respectively. Ctrl, control; DE, differentially expressed. **Fig. S4.** Functional analysis of the differentially expressed mRNAs and lncRNAs. (a) up-regulated (b) and down-regulated DE mRNAs. Gene Ontology analyses. **Fig. S5.** Protein and protein interaction of differentially expressed mRNAs. (a) Top 100 DE mRNAs PPI analysis with high confidence ≥0.7, (b) A significant module with 10 nodes was identified by MCODE (score = 9.556). Red nodes represent up-regulated DE mRNAs and green represent down-regulated DE mRNAs. DE, differentially expressed. **Fig. S6.** Validation of the expression of DE lncRNAs and DE mRNAs by qRT-PCR. (a) expression of lncRNA, (b) expression of mRNA. n=5, * *P *< 0.05 and ** *P* < 0.01. DE, differentially expressed. qRT-PCR, quantitative real-time polymerase chain reaction.

## Data Availability

The dataset supporting the conclusions of this article is available in the Gene Expression Omnibus repository, unique persistent identifier and hyperlink to dataset in https://www.ncbi.nlm.nih.gov/geo/query/acc.cgi?acc=GSE144439*.*

## Abbreviations

ceRNA: Competitive endogenous RNA
CNC: Coding/noncoding gene co-expression
DE: Differentially expressed
GO: Gene ontology
KEGG: Kyoto encyclopedia of genes and genomes
LPS: Lipopolysaccharide
MTFP1: Mitochondrial fission process 1
PPI: Protein–protein interaction
qRT-PCR: Quantitative real-time polymerase chain reaction
MAP: Mean arterial blood pressures
