# Supplementary material for: LncRNA–mRNA expression profile and functional network of vascular dysfunction in septic rats
Source: Eur J Med Res. 2023 Jan 7;28:11. doi: 10.1186/s40001-022-00961-z (PMC9824925; doi:10.1186/s40001-022-00961-z)

##### Additional file

**Table S1.** The detailed information of the top ten up-regulated and top ten down-regulated lncRNAs

**Table S2.** The detailed information of the top ten up-regulated and top ten down-regulated mRNAs

**Table S3.** Primers for LncRNAs validated by qRT-PCR

**Table S4.** Primers for mRNAs validated by qRT-PCR

**Fig. S1 Verify the sepsis model.** (a) Effect of LPS on serum C-reactive protein. Effect of LPS on in vitro vascular (b) contraction reactivity and (c) relaxation responses. n = 4. * *P* < 0.05, significant from control and LPS group. LPS, Lipopolysaccharide.

**Fig. S2 Systematic analysis of significantly differentially expressed lncRNAs and mRNA in arota tissue.** Heat maps showing the expression profiles of (a) lncRNAs and (b) mRNAs; Volcano plots presenting differences in the expression of (c) lncRNAs and (d) mRNAs between the LPS and control groups. Values plotted on the x- and y-axes represent the averaged normalized signal values of each group (log2-scaled). LPS, Lipopolysaccharide.

**Fig. S3** **The distribution of DE lncRNAs and DE mRNAs on chromosomes.** (a, b) Circos plots representing the distribution of DE lncRNAs and DE mRNAs on chromosomes. The outer layer cycle is the chromosome map of the human genome. The inner layers represent the distribution of DE mRNAs and DE lncRNAs on different chromosomes, respectively. Red and green colors represent up and down regulation, respectively. Ctrl, control; DE, differentially expressed.

**Fig. S4** **Functional analysis of the differentially expressed mRNAs and lncRNAs.** (a) up--regulated (b) and down-regulated DE mRNAs. Gene Ontology analyses. DE, differentially expressed.

**Fig. S5 Protein and protein interaction of differentially expressed mRNAs. (**a) Top 100 DE mRNAs PPI analysis with high confidence ≥0.7, (b) A significant module with 10 nodes were identified by MCODE (score = 9.556). Red nodes represent up-regulated DE mRNAs and green represent down-regulated DE mRNAs. DE, differentially expressed.

**Fig. S6 Validation of the expression of DE lncRNAs and DE mRNAs by qRT-PCR**. (a) expression of lncRNA, (b) expression of mRNA. n=5, * *P* < 0.05 and ** *P* < 0.01. DE, differentially expressed. qRT-PCR, quantitative real-time polymerase chain reaction.

**Table S1.** The detailed information of the top ten up-regulated and top ten down-regulated lncRNAs

| **Probe Name** | **Transcript ID** | **P-value** | **Fold Change** | **Regulation** | **chrom** | **Gene Symbol** | **RNA length** |
| --- | --- | --- | --- | --- | --- | --- | --- |
| ASRNV20032326V3 | XR_592112 | 7.84928E-07 | 14.0 | up | chr4 | LOC102546951 | 1779 |
| ASRNV20028780V3 | XR_349314 | 4.40375E-05 | 9.0 | up | chr9 | LOC102555426 | 2358 |
| ASRNV20035412V3 | XR_596552 | 0.000444309 | 7.3 | up | chr16 | LOC103694005 | 5722 |
| ASRNV20029779V3 | XR_362127 | 4.1035E-05 | 7.3 | up | chr20 | LOC102548205 | 2545 |
| ASRNV20034443V3 | XR_594785 | 0.01434769 | 5.8 | up | chr15 | LOC102547310 | 546 |
| ASRNV20032789V3 | XR_592660 | 0.002470748 | 5.5 | up | chr5 | LOC103692427 | 2960 |
| ASRN6LNC1A100001625V3 | ENSRNOT00000089798 | 0.000140202 | 5.0 | up | chr6 | AABR07064635.1 | 417 |
| ASRNV20028808V3 | XR_349493 | 0.000607758 | 4.2 | up | chr2 | LOC102556643 | 279 |
| ASRN6LNC1A100000947V3 | ENSRNOT00000077237 | 9.75768E-06 | 4.2 | up | chr6 | AABR07065530.1 | 306 |
| ASRNV20029280V3 | XR_355499 | 0.000354279 | 3.5 | up | chr7 | LOC102549714 | 519 |
| ASRN6LNC1A100027144V3 | ENSRNOT00000086932 | 0.002977343 | 4.8 | down | chr1 | AABR07001210.1 | 584 |
| ASRN6LNC1A100005564V3 | ENSRNOT00000083801 | 0.011441272 | 4.7 | down | chr18 | AABR07031526.1 | 618 |
| ASRN6LNC1A100001672V3 | ENSRNOT00000083119 | 0.002410818 | 4.0 | down | chr7 | AC141997.1 | 2600 |
| ASRNV20030205V3 | XR_589510 | 0.029284109 | 3.9 | down | chr9 | LOC103690664 | 1799 |
| ASRN6LNC1A100002652V3 | ENSRNOT00000079370 | 0.013060872 | 3.9 | down | chr7 | AABR07057628.1 | 439 |
| ASRNV20035815V3 | XR_597034 | 0.012228722 | 3.8 | down | chr18 | LOC103694232 | 351 |
| ASRN6LNC1A100005915V3 | ENSRNOT00000079309 | 0.020367365 | 3.6 | down | chr14 | AABR07015705.1 | 663 |
| ASRNV20030598V3 | XR_590074 | 0.002431442 | 3.5 | down | chr1 | LOC102548473 | 627 |
| ASRNV20027970V3 | XR_344417 | 0.017788084 | 3.3 | down | chr2 | LOC102550008 | 556 |
| ASRNV20026821V3 | XR_086340 | 0.002029692 | 2.9 | down | chr9 | LOC301444 | 328 |

**Table S2.** The detailed information of the top ten up-regulated and top ten down-regulated mRNAs

| **Probe Name** | **Transcript_ID** | **P-value** | **Fold Change** | **Regulation** | **chrom** | **Gene Symbol** | **RNA length** |
| --- | --- | --- | --- | --- | --- | --- | --- |
| ASRN6AP1B100050044V3 | ENSRNOT00000073796 | 0.00133364 | 19.1 | down | chr6 | Adssl1 | 1797 |
| ASRN6AP1B100083221V3 | ENSRNOT00000078571 | 0.00088096 | 5.0 | down | chr8 | Cib2 | 1408 |
| ASRN6AP1B100026850V3 | ENSRNOT00000019679 | 0.00192379 | 7.4 | down | chr1 | Cox8b | 319 |
| ASRN6AP1B100008547V3 | ENSRNOT00000017325 | 0.00482482 | 16.1 | down | chr5 | Fabp3 | 763 |
| ASRN6AP1B100014982V3 | ENSRNOT00000005768 | 8.898E-06 | 23.8 | down | chr13 | Lrrc52 | 1421 |
| ASRN6AP1B100014985V3 | ENSRNOT00000006160 | 0.00054643 | 6.2 | down | chr14 | Mtfp1 | 1362 |
| ASRN6AP1B100047894V3 | ENSRNOT00000009683 | 0.00020504 | 17.8 | down | chr5 | Nr0b2 | 1111 |
| ASRN6AP1B100030437V3 | ENSRNOT00000018447 | 0.00584211 | 12.5 | down | chr1 | Scd | 4475 |
| ASRN6AP1B102485728V3 | ENSRNOT00000092278 | 9.7713E-05 | 21.1 | down | chr1 | Shank2 | 1061 |
| ASRN6AP1B100029412V3 | ENSRNOT00000004900 | 3.5713E-06 | 10.0 | down | chr19 | Ucp1 | 1198 |
| ASRN6AP1B101943841V3 | ENSRNOT00000073042 | 6.2085E-07 | 51.1 | up | chr9 | C3 | 5188 |
| ASRN6AP1B100018098V3 | ENSRNOT00000000256 | 7.6571E-07 | 107.5 | up | chr10 | Ccl7 | 850 |
| ASRN6AP1B100051090V3 | ENSRNOT00000003778 | 1.3897E-08 | 105.8 | up | chr14 | Cxcl1 | 926 |
| ASRN6AP1B100044615V3 | ENSRNOT00000013732 | 1.2807E-07 | 76.0 | up | chr4 | Il6 | 1045 |
| ASRN6AP1B102443765V3 | ENSRNOT00000058479 | 1.8267E-07 | 162.6 | up | chr11 | Kng1 | 1299 |
| ASRN6AP1B100084666V3 | ENSRNOT00000018776 | 1.2357E-06 | 46.0 | up | chr3 | Lcn2 | 889 |
| ASRN6AP1B100029814V3 | ENSRNOT00000010454 | 4.5866E-07 | 60.3 | up | chr5 | Orm1 | 1020 |
| ASRN6AP1B100012079V3 | ENSRNOT00000015473 | 9.2652E-05 | 36.4 | up | chr2 | S100a8 | 377 |
| ASRN6AP1B100001211V3 | ENSRNOT00000015351 | 0.00010394 | 20.8 | up | chr2 | S100a9 | 528 |
| ASRN6AP1B100060870V3 | ENSRNOT00000054932 | 3.8726E-07 | 42.0 | up | chr10 | Sectm1b | 1160 |

**Table S3.** Primers for LncRNAs validated by qRT-PCR

| **Gene** | **Forward and Reverse primer sequence** |
| --- | --- |
| GAPDH(RAT) | F:5’ GCTCTCTGCTCCTCCCTGTTCTA3'  R:5’ TGGTAACCAGGCGTCCGATA3’ |
| XR_592112 | F:5' GTCTGTCCCCTCACTTCTGTC 3’  R:5’ TAGCCCCATCACTTAGCATTA 3’ |
| XR_349314 | F:5' ACTCATTGGTCGCTCCTTACT 3’  R:5’ CTGTGTCCTGGGGGTAGATT 3’ |
| XR_596552 | F:5' GTAAGTAAGAGGTCATTGCCCA 3’  R:5’ TTTCCTCTCCAACCCTCTATG 3’ |
| XR_362127 | F:5' TATTTTCCACATCTGATTTCCG 3’  R:5’ CGCACATACCTCTTTCGTTTC 3’ |
| XR_594785 | F:5' GGAACATTTATTAATGGTGTGTATT3’  R:5’ CCAAAGACACACGTTAGACCTG 3’ |
| XR_592660 | F:5' CTCCCTAAAATCTGACGCTG3’  R:5’ ATCTCTCTGAACTCCAACTTCC 3’ |
| ENSRNOT00000089798 | F:5' TGTCTTGAACTTGTTACCATCC 3’  R:5’ TGAGAAGGTTGCTGTAGTCCC 3’ |
| XR_349493 | F:5' CCACAGCCCTGATTTTACTGTG 3’  R:5’ TCTCTCCAAACGGCTCTCTG 3’ |
| ENSRNOT00000077237 | F:5' TGCCTGGGTAGAAATAAGACAC 3’  R:5’ ATCCATCATTACCTGCCACA 3’ |
| ENSRNOT00000086932 | F:5' ATGAAGTGGATGCATTCAGGTCT 3’  R:5’ CATGGAACGAAAGGTGGCAA 3’ |
| ENSRNOT00000083801 | F:5' CCTTCTCCTGGTTCTGGTATTA 3’  R:5’ CATCTTTTCTCAACAGAGTCTCAC 3’ |
| ENSRNOT00000083119 | F:5' TGATCAAAGTACATTGGACCGG 3’  R:5’ CTTCGTTATTTTGGAACCCGTT 3’ |
| XR_589510 | F:5' TGAATGCTGTCTGGGGCTAAC 3’  R:5’ GCTGTTGGCACATTCTGATCTT 3’ |
| ENSRNOT00000079370 | F:5' TTGAGTGAAGAGAAGTCTGGCA 3’  R:5’ CGTGATTTTTGAAACTGAGTGTG 3’ |
| XR_597034 | F:5' GGCTTCCGCTTTCCTGTTA 3’  R:5’ GCTGCCTGATCTTCTATCTACCA 3’ |
| ENSRNOT00000079309 | F:5' TCTGAAACTGCCAGGAAGAA 3’  R:5’ GGTGCTGATTTGGTACTAAAGG 3’ |
| XR_590074 | F:5' TGCTAGGGCTACAGGTGTAAGC 3’  R:5’ GAGATAGAAGGGGGAATGGGAC 3’ |
| XR_355499 | F:5' GCCATACAATGTGCGAAGAGAAG 3’  R:5’ GTTTGAGGGGAAAGCAAGGAAG 3’ |
| XR_344417 | F:5' AAGCTAACATCCCACTACATGC3’  R:5’ GGTAGCCCAAGAGGAAATCA 3’ |
| XR_086340 | F:5' CTGTGGGTGACATAAATACAGATA 3’  R:5’ CTCTTTTACCCTTTTCCACATA 3’ |

**Table S4.** Primers for mRNAs validated by qRT-PCR

| **Gene** | **Forward and Reverse primer sequence** |
| --- | --- |
| GAPDH(RAT) | F:5’ GCTCTCTGCTCCTCCCTGTTCTA3'  R:5’ TGGTAACCAGGCGTCCGATA3’ |
| Kng1 | F:5’ TTTTTGGCAAAACAATTCCTCA 3’  R:5’ GCAGTCCATTTCCTGGGCA 3’ |
| Ccl7 | F:5’ CGCTTCTGTGTGTGCTGCTCA 3’  R:5’ GCCTCCTCAACCCACTTCTG 3’ |
| Cxcl1 | F:5’ GTGTTTTGTGTTAGGGTGAGG 3’  R:5’ GACGAGAAGGAGCATTGGTTA 3’ |
| Il6 | F:5’ TCAGAGCAATACTGAAACCCTA 3’  R:5’ TCCTTAGCCACTCCTTCTGT 3’ |
| Orm1 | F:5’ ACTTCGGGAGTTTCAGACCAC 3’  R:5’ CTTCAGCACTATCAAATGGGC 3’ |
| C3 | F:5’ TGGACCATAGAAGAGTTGAA 3’  R:5’ CACAGAGTAGGGCAGTCG 3’ |
| Lcn2 | F:5’ CGTCACTTCCATCCTCGTCA 3’  R:5’ CTGGTCGTAGTCAGTGTCGG 3’ |
| S100a8 | F:5’ GGGAATCACCATGCCCTCTAC 3’  R:5’ GCCCACCCTTATCACCAACAC 3’ |
| S100a9 | F:5’ GGCACGAGCTCCTTAGCTTT 3’  R:5’ GGTTTGTGTCCAGGTCCTCC 3’ |
| Lrrc52 | F:5’ AGTGGAAACCCGTGGAAGTG 3’  R:5’ CTCCGTACACGTGGCATTCT 3’ |
| Shank2 | F:5’ TACCCCCATTGAGGAATTCAC 3’  R:5’ CAATCAAGAAGTCCCCGGTC 3’ |
| Adssl1 | F:5’ TGACCACGGGCAGGAAGA 3’  R:5’ GTTAGCAGGGAAGTAGGGGAT 3’ |
| Nr0b2 | F:5’ GGCACTATCCTCTTCAACCCA 3’  R:5’ GGAAGCCATGAGGAGGATTCG 3’ |
| Fabp3 | F:5’ AGGTGGCTAGCATGACCAAG 3’  R:5’ GTCCCACTTCTGCACATGGA 3’ |
| Scd | F:5’ ACATCCGTCCTGAAATGAGA 3’  R:5’ AGGGCACTGATAAGGTAGTAAA 3’ |
| Ucp1 | F:5’ GCCTAGCAGACATCATCACCT 3’  R:5’ CCAGCCGAGATCTTGCTTCC 3’ |
| Cox8b | F:5’ CACTTCTGCCATGGATCAGG 3’  R:5’ GGAACCATGAAGCCAGCGAT 3’ |
| Mtfp1 | F:5’ TGTCATTGTCCACCCCATTGA 3’  R:5’ GAGCCACGTAGCCATGTTCTT 3’ |
| Cib2 | F:5’ TACCAGGACTGCACTTTCTTCA 3’  R:5’ ACGATGGGGCTCTTCCTGTA 3’ |
| Sectm1b | F:5’ CTCCAGGAAACTACTCTAAGGAC 3’  R:5’ CTGGTTGTCAGTGGCTAGACC 3’ |

**Fig. S1 Verify the sepsis model.** (a) Effect of LPS on serum C-reactive protein. Effect of LPS on in vitro vascular (b) contraction reactivity and (c) relaxation responses. n = 4. * *P* < 0.05, significant from control and LPS group. LPS, Lipopolysaccharide.

**

**

**Fig. S2 Systematic analysis of significantly differentially expressed lncRNAs and mRNA in arota tissue.** Heat maps showing the expression profiles of (a) lncRNAs and (b) mRNAs; Volcano plots presenting differences in the expression of (c) lncRNAs and (d) mRNAs between the LPS and control groups. Values plotted on the x- and y-axes represent the averaged normalized signal values of each group (log2-scaled). LPS, Lipopolysaccharide.


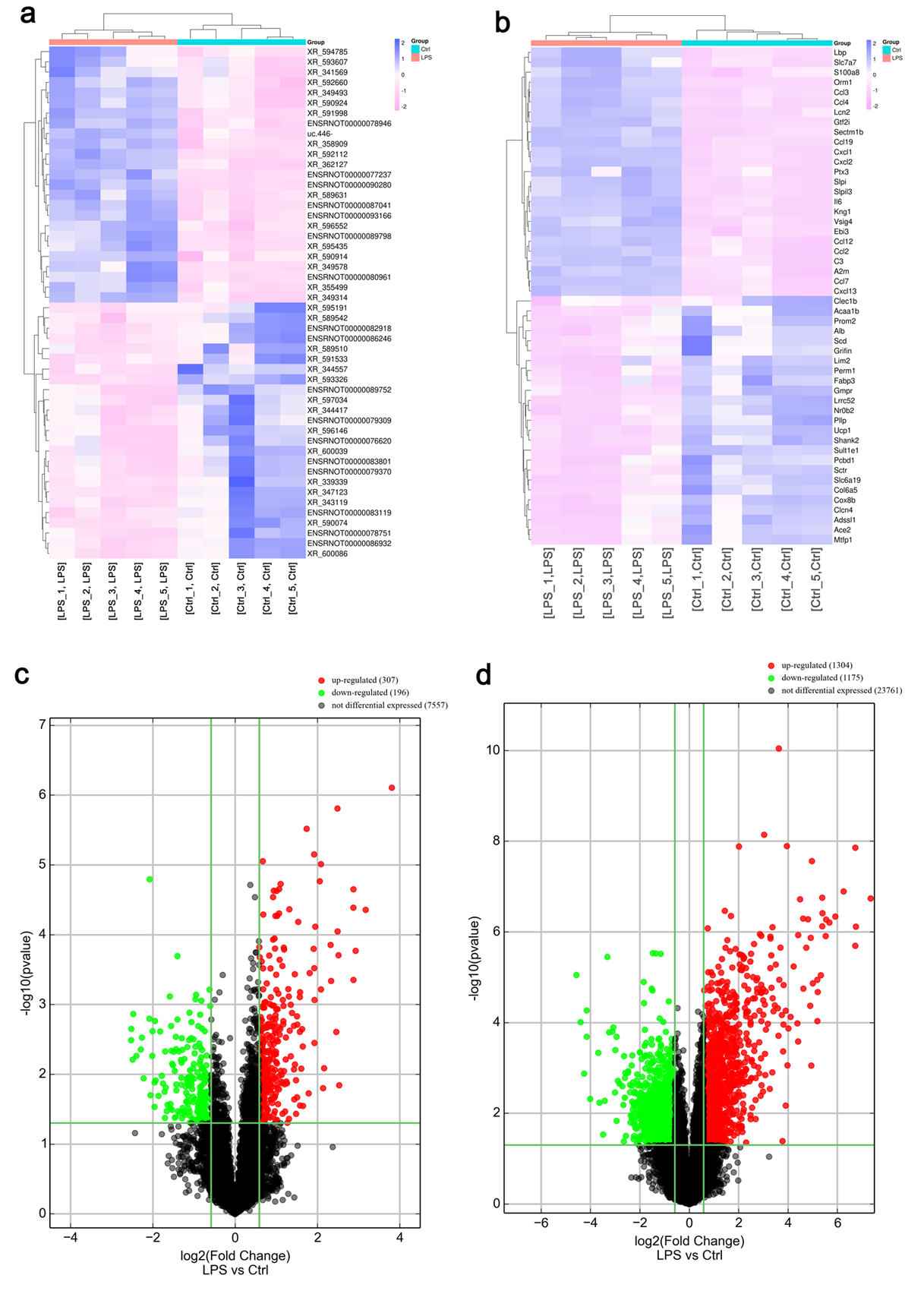


**Fig. S3** **The distribution of DE lncRNAs and DE mRNAs on chromosomes.** (a, b) Circos plots representing the distribution of DE lncRNAs and DE mRNAs on chromosomes. The outer layer cycle is the chromosome map of the human genome. The inner layers represent the distribution of DE mRNAs and DE lncRNAs on different chromosomes, respectively. Red and green colors represent up and down regulation, respectively. Ctrl, control; DE, differentially expressed.


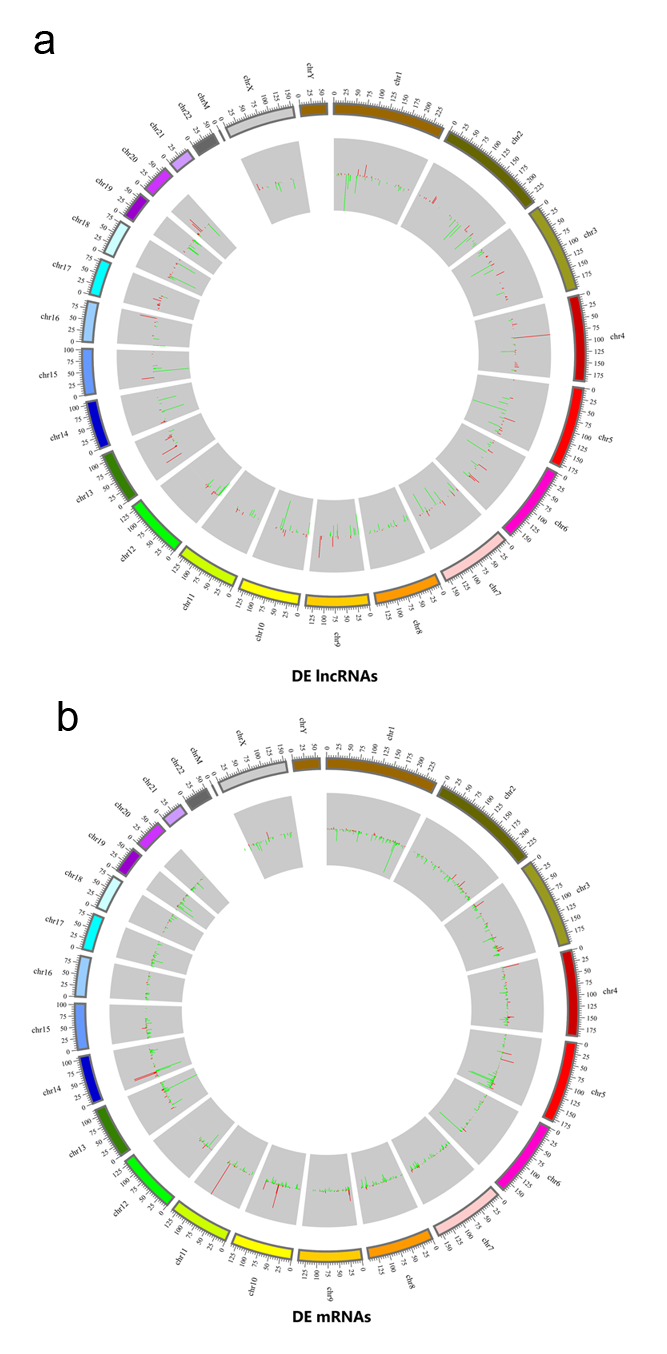


**Fig. S4** **Functional analysis of the differentially expressed mRNAs and lncRNAs.** (a) up--regulated (b) and down-regulated DE mRNAs; Gene Ontology analyses. DE, differentially expressed.


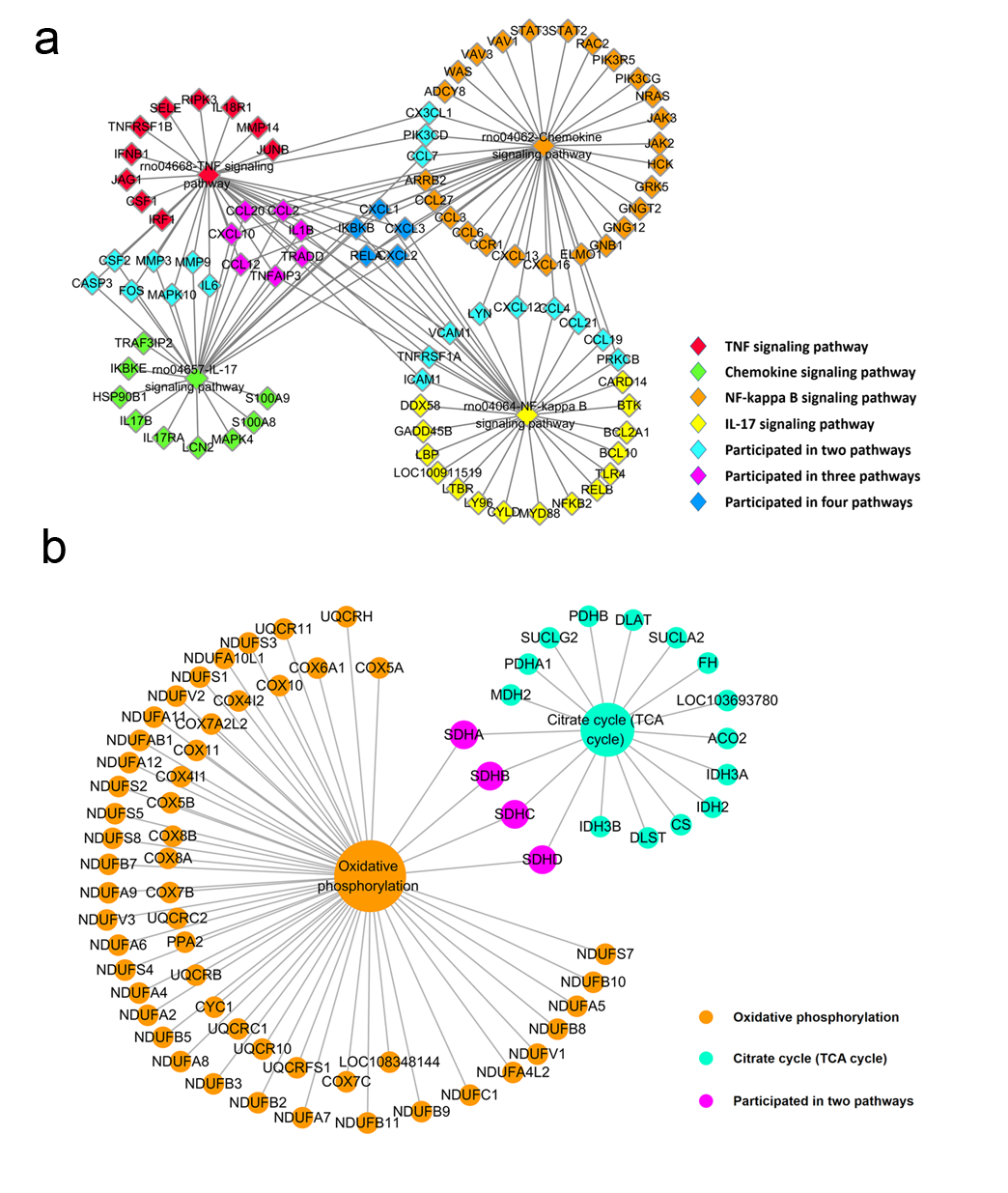


**Fig. S5 Protein and protein interaction of differentially expressed mRNAs. (**a) Top 100 DE mRNAs PPI analysis with high confidence ≥0.7, (b) A significant module with 10 nodes were identified by MCODE (score = 9.556). Red nodes represent up-regulated DE mRNAs and green represent down-regulated DE mRNAs. DE, differentially expressed.


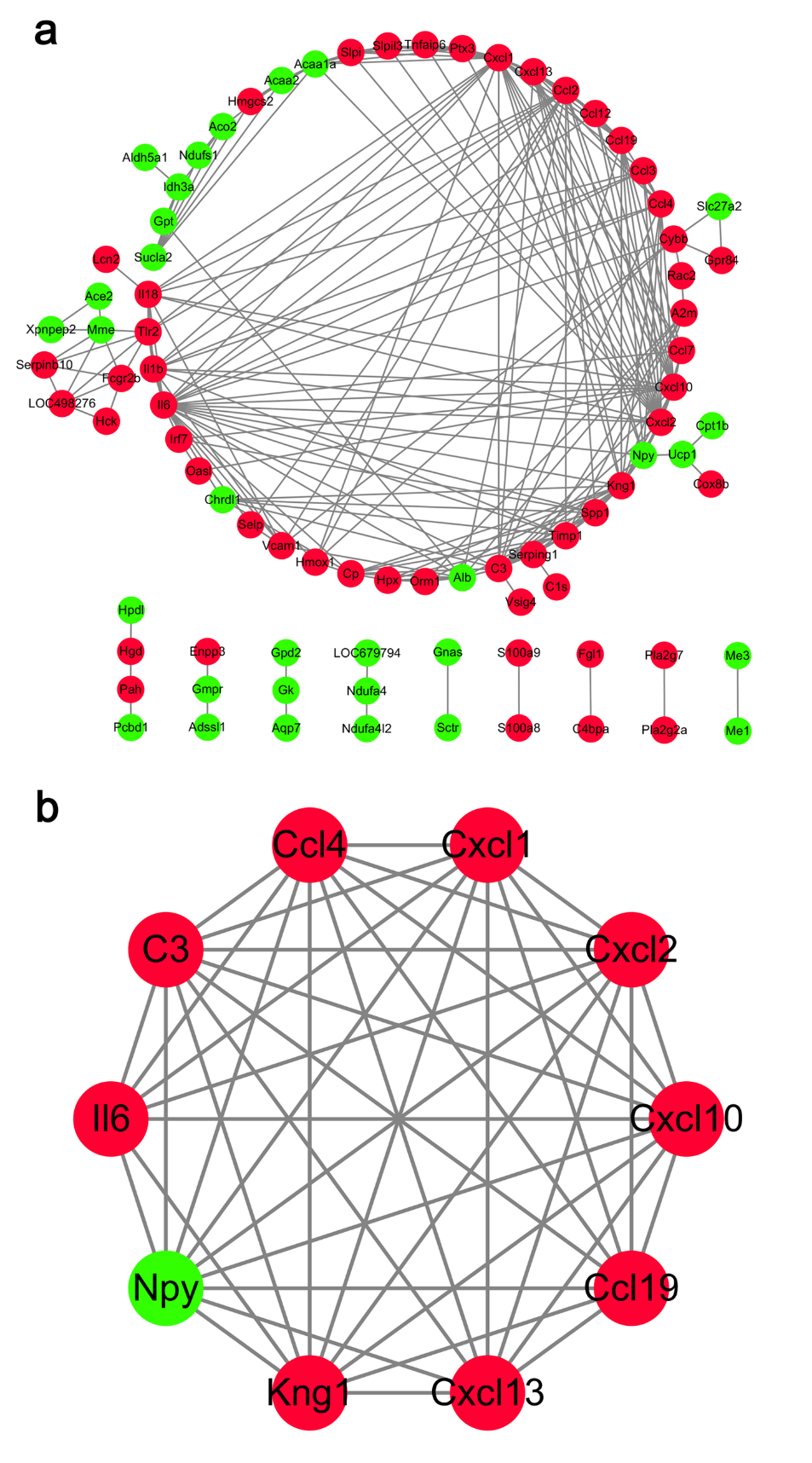


**Fig. S6 Validation of the expression of DE lncRNAs and DE mRNAs by qRT-PCR**. (a) expression of lncRNA, (b) expression of mRNA. n=5, * *P* < 0.05 and ** *P* < 0.01. DE, differentially expressed. qRT-PCR, quantitative real-time polymerase chain reaction.


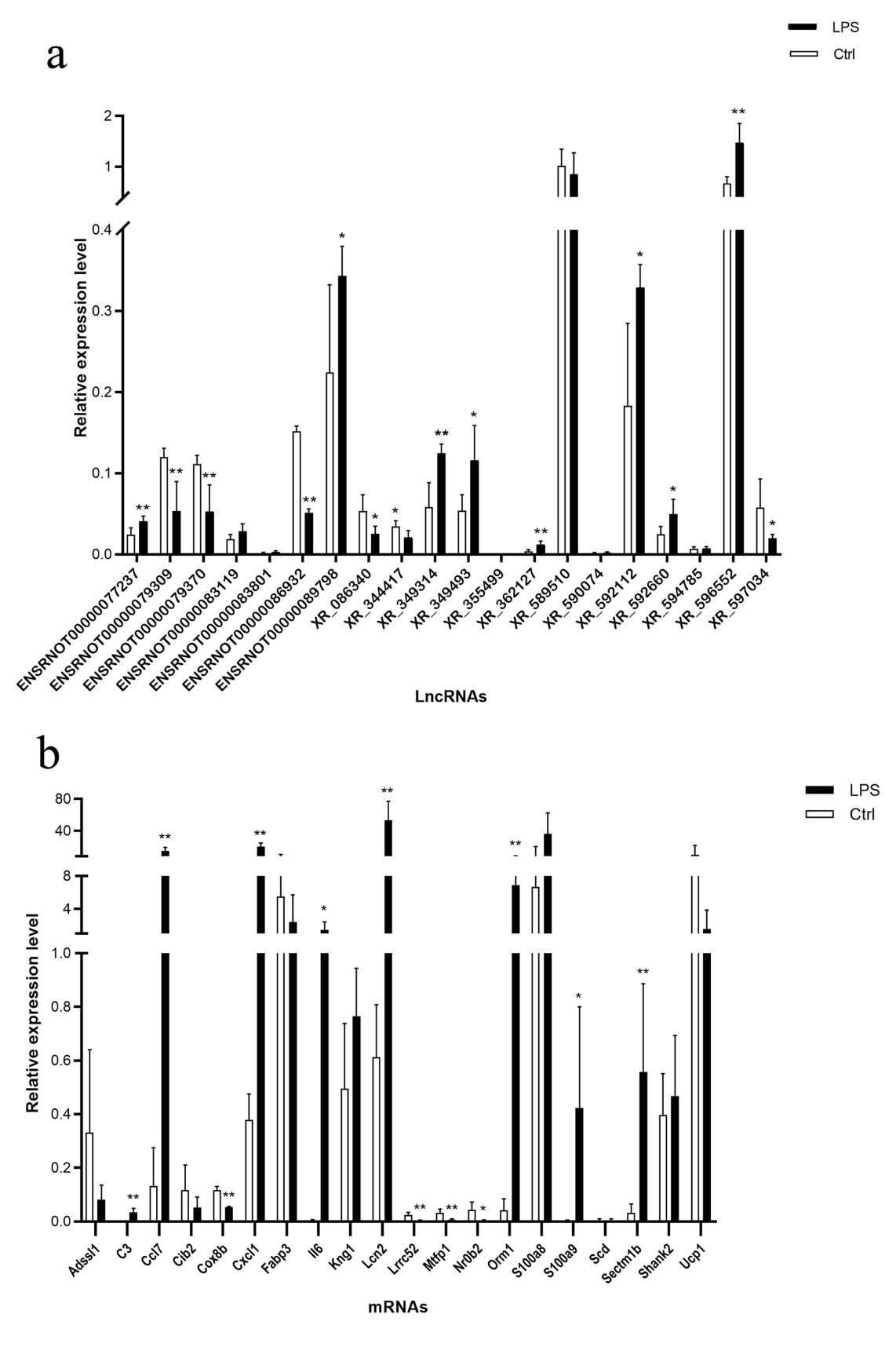

Supplement: Supplementary file 1 — Additional file 1: Table S1. The detailed information of the top ten up-regulated and top ten down-regulated lncRNAs. Table S2. The detailed information of the top ten up-regulated and top ten down-regulated mRNAs. Table S3. Primers for LncRNAs validated by qRT-PCR. Table S4. Primers for mRNAs validated by qRT-PCR. Fig. S1. Verify the sepsis model. (a) Effect of LPS on serum C-reactive protein. Effect of LPS on in vitro vascular (b) contraction reactivity and (c) relaxation responses. n = 4. * P < 0.05, significant from the control and LPS group. LPS, Lipopolysaccharide. Fig. S2. Systematic analysis of significantly differentially expressed lncRNAs and mRNA in aorta tissue. Heat maps showing the expression profiles of (a) lncRNAs and (b) mRNAs; Volcano plots presenting differences in the expression of (c) lncRNAs and (d) mRNAs between the LPS and control groups. Values plotted on the x- and y-axes represent the averaged normalised signal values of each group (log2-scaled). LPS, Lipopolysaccharide. Fig. S3. The distribution of DE lncRNAs and DE mRNAs on chromosomes. (a, b) Circos plots representing the distribution of DE lncRNAs and DE mRNAs on chromosomes. The outer layer cycle is the chromosome map of the human genome. The inner layers represent the distribution of DE mRNAs and DE lncRNAs on different chromosomes, respectively. Red and green colors represent up- and down-regulation, respectively. Ctrl, control; DE, differentially expressed. Fig. S4. Functional analysis of the differentially expressed mRNAs and lncRNAs. (a) up-regulated (b) and down-regulated DE mRNAs. Gene Ontology analyses. Fig. S5. Protein and protein interaction of differentially expressed mRNAs. (a) Top 100 DE mRNAs PPI analysis with high confidence ≥0.7, (b) A significant module with 10 nodes was identified by MCODE (score = 9.556). Red nodes represent up-regulated DE mRNAs and green represent down-regulated DE mRNAs. DE, differentially expressed. Fig. S6. Validation of the expression of DE ln [file 40001_2022_961_MOESM1_ESM.docx]
